# Humanized Anti-CD19 CAR-T Cell Therapy and Sequential Allogeneic Hematopoietic Stem Cell Transplantation Achieved Long-Term Survival in Refractory and Relapsed B Lymphocytic Leukemia: A Retrospective Study of CAR-T Cell Therapy

**DOI:** 10.3389/fimmu.2021.755549

**Published:** 2021-10-29

**Authors:** Wei Chen, Yuhan Ma, Ziyuan Shen, Huimin Chen, Ruixue Ma, Dongmei Yan, Ming Shi, Xiangmin Wang, Xuguang Song, Cai Sun, Jiang Cao, Hai Cheng, Feng Zhu, Haiying Sun, Depeng Li, Zhenyu Li, Junnian Zheng, Kailin Xu, Wei Sang

**Affiliations:** ^1^ Department of Hematology, The Affiliated Hospital of Xuzhou Medical University, Xuzhou, China; ^2^ Department of Hematology, The First People’s Hospital of Suqian, Suqian, China; ^3^ Blood Diseases Institute, Xuzhou Medical University, Xuzhou, China; ^4^ Department of Epidemiology and Biostatistics, School of Public Health, Xuzhou Medical University, Xuzhou, China; ^5^ Cancer Institute, Xuzhou Medical University, Xuzhou, China; ^6^ Jiangsu Center for the Collaboration and Innovation of Cancer Biotherapy, Cancer Institute, Xuzhou Medical University, Xuzhou, China; ^7^ Center of Clinical Oncology, The Affiliated Hospital of Xuzhou Medical University, Xuzhou, China

**Keywords:** chimeric antigen receptor T cell therapy (CAR-T), hematopoietic stem cell transplantation, relapsed/refractory B cell lymphoblastic leukemia, overall survival, leukemia free survival, minimal residual disease (MRD)

## Abstract

Early response could be obtained in most patients with relapsed or refractory B cell lymphoblastic leukemia (R/R B-ALL) treated with chimeric antigen receptor T-cell (CAR-T) therapy, but relapse occurs in some patients. There is no consensus on treatment strategy post CAR-T cell therapy. In this retrospective study of humanized CD19-targeted CAR-T cell (hCART19s) therapy for R/R B-ALL, we analyzed the patients treated with allogeneic hematopoietic stem cell transplantation (allo-HSCT) or received a second hCART19s infusion, and summarized their efficacy and safety. We retrospectively studied 28 R/R B-ALL patients treated with hCART19s in the Affiliated Hospital of Xuzhou Medical University from 2016 to 2020. After the first hCART19s infusion, 10 patients received allo-HSCT (CART+HSCT group), 7 patients received a second hCART19s infusion (CART2 group), and 11 patients did not receive HSCT or a second hCART19s infusion (CART1 group). The safety, efficacy, and long-term survival were analyzed. Of the 28 patients who received hCART19s treatment, 1 patient could not be evaluated for efficacy, and 25 (92.6%) achieved complete remission (CR) with 20 (74.7%) achieving minimal residual disease (MRD) negativity. Seven (25%) patients experienced grade 3-4 CRS, and one died from grade 5 CRS. No patient experienced ≥3 grade ICANS. The incidence of second CR is higher in the CART+HSCT group compared to the CART2 group (100% *vs.* 42.9%, p*=*0.015). The median follow-up time was 1,240 days (range: 709–1,770). Significantly longer overall survival (OS) and leukemia-free survival (LFS) were achieved in the CART+HSCT group (median OS and LFS: not reached, p*=*0.006 and 0.001, respectively) compared to the CART2 group (median OS: 482; median LFS: 189) and the CART1 group (median OS: 236; median LFS: 35). In the CART+HSCT group, the incidence of acute graft-*versus*-host disease (aGVHD) was 30% (3/10), and transplantation-related mortality was 30% (3/10). No chronic GVHD occurred. Multivariate analysis results showed that blasts ≥ 20% in the bone marrow and MRD ≥ 65.6% are independent factors for inferior OS and LFS, respectively, while receiving allo-HSCT is an independent factor associated with both longer OS and LFS. In conclusion, early allo-HSCT after CAR-T therapy can achieve long-term efficacy, and the adverse events are controllable.

## Introduction

Patients with relapsed or refractory (R/R) acute B-cell lymphoblastic leukemia (B-ALL) progress rapidly, and the overall 5-year survival rate is only 10–20% (1, 2). The response rate of salvage chemotherapy is low. At present, allogeneic hematopoietic stem cell transplantation (allo-HSCT) is the only cure for patients with R/R B-ALL. However, only a few patients have the opportunity to undergo allo-HSCT.

As a novel therapeutic strategy, rapid progress has been made in chimeric antigen receptor T-cell (CAR-T) therapy in recent years, especially in hematological malignancies, which showed a high remission rate (3, 4). We previously reported that the effective rate of CAR-T cell therapy targeting CD19 was 93% in R/R B-ALL patients (5, 6). These findings were consistent with those of Davila et al. (3), who reported that the complete remission (CR) rate of CD19 CAR-T on R/R B-ALL can reach 88%, and the adverse events can be tolerated. On the other hand, CAR-T cell therapy has shown significant therapeutic efficacy in turning MRD negativity. A few large-sale studies (7, 8) also indicated that CAR-T cell therapy promoted negative MRD (range: 67%–87%). Notably, pre-transplantation status, especially MRD status, is related to long-term survival after HSCT (9, 10).

Although a high MRD-negative CR rate was achieved after CAR-T therapy, the long-term efficacy was unsatisfactory due to loss of the CAR-T cells resulting from the limited long-term persistence, the immune-suppressive microenvironment, and exhaustion of CAR-T cells (11, 12). It is necessary to optimize the strategy of treatment to further improve long-term efficacy after CAR-T cell infusion. However, either bridging HSCT or second CART is controversial in improving long-term efficacy. Gauthier et al. (13) noted that only 21% of patients obtained CR after second infusion of anti-CD19 CAR-T cells, while the median duration of response was merely 4 months. On the other hand, Zhang et al. (14) demonstrated that 184 patients who underwent allo-HSCT had better 2-year OS and LFS than patients who did not (68% *vs.* 28.3, 60.4% *vs.* 27.8%, respectively, p*<*0.001), but lack longer follow-up. This is consistent with the findings of Hay et al., who found that allo-HSCT after anti-CD19 CAR-T cell therapy was associated with a better LFS (15). On the contrary, Park et al. (16) reported that relapse and transplant-related toxicities were the main causes of death for 17 patients who underwent allo-HSCT after CAR-T therapy, suggesting that the patients seemed not to benefit from allo-HSCT.

Therefore, we conducted long-term follow-up and retrospectively analyzed the efficacy and safety of patients who received HSCT or a second hCART19s infusion as a sequential treatment after the first hCART19s infusion.

## Patients and Methods

### Patients

We retrospectively reviewed the data on CD19+ R/R B-ALL patients who received hCART19s therapy at the Affiliated Hospital of Xuzhou Medical University from May 2016 to May 2020. All the enrolled patients had relapsed or refractory disease. The eligibility criteria were age less than 70 years; good organ function and evaluation of a survival longer than 3 months; and Eastern Cooperative Oncology Group (ECOG) performance status <2. All patients provided signed informed consent before the hCART19s therapy and allo-HSCT. Patients received HSCT or a second infusion of hCART19s after hCART19s infusion depending on patients’ choice, disease status, and their affordability to the treatment. Before HSCT, all patients were in MRD-negative CR after the first hCART19s infusion. In some cases of relapse after the first hCART19s infusion, the second hCART19s was infused as soon as the relapse was clinically confirmed (the detail is listed in **Supplementary Table 2**). Patients were divided into three groups: the first infusion of hCART19s without transplant or a second infusion of hCART19s (CART1 group), a second infusion of hCART19s without transplant (CART2 group), and the first infusion of hCART19s followed by HSCT (CART+HSCT group).

The study protocol was approved by the human studies review board at the Affiliated Hospital of Xuzhou Medical University (ClinicalTrials.gov # NCT02782351). The clinical investigation was conducted according to the principles of the Declaration of Helsinki.

### CAR-T Cell Treatment Protocol

HCART19s constructed with 4-1BB costimulatory domain were generated *via* a lentiviral vector as previously reported (6). All the hCART19s required quality control before discharge. After a lymphocyte-depleting chemotherapy with a fludarabine and cyclophosphamide (FC) regimen (fludarabine at 30 mg/m^2^ per day for 3 days and cyclophosphamide at 300 mg/m^2^ per day for 3 days), all patients provided signed informed consent before the hCART19s therapy and allo-HSCT and received a single dose of autologous hCART19s infusion at 1×10^6^ CAR-T cells/kg (**Supplementary Table 1**). No patient experienced bridged chemotherapy from preparation of CAR-T cells to infusion, and lymphodepletion was also conducted prior to the second CAR T infusion.

### Transplant Protocol

All patients received marrow ablative regimens using BU/CY or modified BU/CY strategies for matched related transplantation, and anti-thymocyte globulin (ATG, rabbit) was administered for haploidentical or unrelated transplantation as a prophylactic against graft-*versus*-host disease (GVHD). Prophylactic regimens of GVHD were determined by the individual transplant physician based upon disease-related and transplant-related considerations composed of cyclosporine, methotrexate, and mycophenolate mofetil.

### Assessment of Toxicity

The cytokine release syndrome was graded according to the cytokine release syndrome grading system. The cytokine release syndrome was considered to be severe if it was of grade 3 or higher. Neurotoxic effects were assessed according to the National Cancer Institute Common Terminology Criteria for Adverse Events, version 4.03. Severe neurotoxic effects were defined as a seizure of any grade or a toxic effect of grade 3 or higher.

### Assessment of Response

Response to therapy was assessed using morphological analysis and multicolor flow cytometry. CR was defined as less than 5% bone marrow blasts, the absence of circulating blasts, and no extramedullary sites of disease (as assessed by means of computed tomography or positron-emission tomography), regardless of cell count recovery. MRD negativity was defined as less than 0.01% bone marrow blasts for all samples analyzed by multicolor flow cytometry. MRD detection was performed at 1, 2, 3, 6, 12, 18, 24, 36, and 48 months after hCART19s therapy or HSCT. Relapsed disease was defined as the reappearance of blasts in blood or bone marrow or in an extramedullary site after a CR. Overall survival (OS) was defined as the time from infusion to the date of death from any cause. Leukemia-free survival (LFS) was calculated from the date of CR to the date of relapse, death, or the last follow-up.

### Statistical Analyses

All measurement data were described using median and range and compared using Mann–Whitney U tests. Enumeration data were presented as frequency (%) and compared using chi-square tests or Fisher’s exact test. Follow-up time was estimated using the Kaplan–Meier method, whereas OS and LFS were estimated using the Kaplan–Meier method. A Cox regression model was used to obtain the hazard ratio (HR) estimates and corresponding 95% confidence intervals (CIs) for OS and LFS. The X-tile 3.6.1 software was used to determine the optimal cutoff values for MRD. All tests were two-sided, and p*<* 0.05 was considered statistically significant. Data were analyzed using SPSS version 26.0 and Graphpad Prism version 8.

## Results

### Patient Characteristics

From May 2016 to May 2020, a total of 28 patients were enrolled in this study, including 12 males and 16 females, with a median age of 22 years. The characteristics of the patients are shown in **Table 1**, and detailed information is listed in **Supplementary Table 1**. There was no statistical difference among the baseline data of the three groups.

**Table 1:** Patient characteristics.

Characteristics	CART+HSCT	CART2	CART1	*P*		
	(n = 10)	(n = 7)	(n = 11)	a	b	c
Age, years				0.291	0.807	0.687
Median (range)	19 (6-54)	8 (6-68)	33 (5-70)			
Sex: Male, n (%)	5 (50)	4 (57)	3 (27)	0.581	0.268	0.22
BCR-ABL1, n (%)	2 (20)	1 (14)	2 (18)	0.64	0.669	0.674
Prior intensive therapies				0.475	0.349	0.930
Median (range)	4 (2-11)	7 (2-17)	6 (2-16)			
Primary refractory to chemotherapy, n (%)	1 (10.0)	1 (14)	2 (18.2)	1	1	1
Number of relapses						
Median (range)	1 (1-2)	1 (1-2)	1 (1-3)	0.864	0.4	0.529
MRD at infusion				0.27	0.349	0.659
Median (range)%	15.7 (0.1-71.9)	36.7 (10-72.2)	29.7 (0.1-96.9)			
BM blasts before CAR-T				0.421	0.654	0.724
Median (range)%	14 (0-86)	42 (0-95)	18 (0-92)			
Volume of CAR-T cells				0.699	0.918	0.724
Median (range)	50 (50-100)	50 (20-100)	50 (50-100)			
Time from CAR-T to last chemotherapy				0.949	0.659	0.637
≤3months	7	5	6			
>3months	3	2	5			

CART, Chimeric Antigen Receptor T-Cell (CAR-T) therapy; CART+HSCT group, patients who received allogeneic hematopoietic stem cell transplantation after CAR-T; CART2 group, patients who received a second hCART19s infusion after CAR-T; CART1 group, patients who did not receive HSCT or a second hCART19s infusion; MRD, minimal residual disease; BM, bone marrow; a=CART+HSCT group vs. CART2 group; b= CART+HSCT group vs. CART1 group; c=CART2 group vs. CART1 group.

### Efficacies of CAR-T Cell Therapy

**Figure 1:**
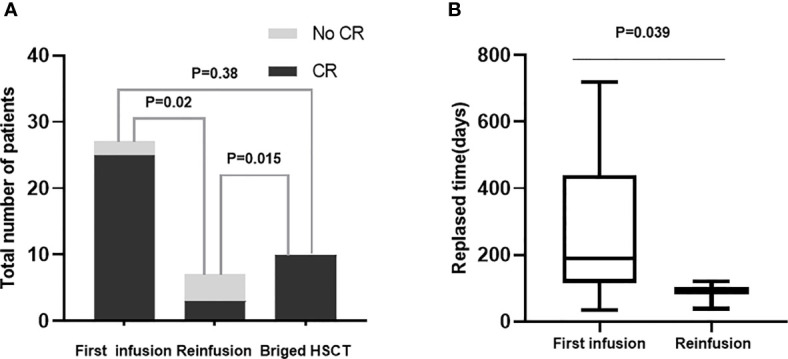
**(A)** The influence of different times of infusion and receving allo-HSCT on CR rate. First infusion (n = 27); Reinfusion (n = 7); Bridge HSCT (n = 10). **(B)** The relapsed days according to times of infusion. First infusion (n = 14); Reinfusion (n = 3).

Of the 28 patients receiving hCART19s infusion, 27 patients were evaluated for response at 14 or 28 days, and 1 patient died at day 14 before being evaluated. The complete remission rate was 92.6% (25/27), with 20 patients having MRD negativity. The rates of CR did not differ significantly among the aforementioned three groups after the first hCART19s infusion. Seven patients received a second hCART19s infusion, three of whom achieved second CR with MRD negativity (42.9%). Notably, as shown in **Figure 1**, compared to the first infusion, the proportion of patients with CR is lower after second infusion (92.6% *vs.* 42.9%, respectively, p*=*0.02).

### CAR-T Cell Toxicities

**Figure 2:**
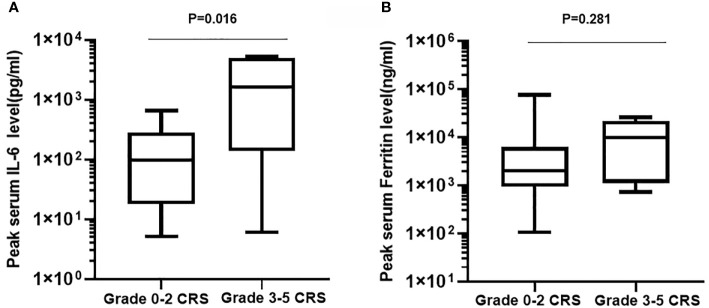
Peak serum levels of IL-6 **(A)** and ferritin **(B)** in patients who developed grade 3-5 CRS (n=8) compared with those with grade 0-2 CRS (n=20).

CRS was the most common nonhematological adverse event after the infusion of hCART19s, which occurred in 27 of the 28 patients, including 8 patients experiencing severe CRS. The median time of CRS occurrence was 6 days (range: 1–20). Patients with CRS were treated with nonsteroidal anti-inflammatory drugs, glucocorticoids, and tocilizumab. One patient died from grade 5 CRS at day 14 after infusion of hCART19s, and one patient died from encephalorrhagia at 24 days after infusion of hCART19s. The peak serum levels of IL-6 in patients who developed grade 3–5 CRS were higher than those with grade 0–2 CRS (n=20). There was no statistical difference in serum ferritin levels between patients with grade 0–2 CRS and grade 3–5 CRS (**Figure 2**). Neurotoxicity occurred in one patient in the CART+HSCT group, with symptoms of convulsion. In addition, all the patients developed B cell dysfunction, manifested as CD19+ B cell deletion and hypogammaglobulinemia. III–IV hematological toxicity and other adverse events are shown in **Table 2**.

**Table 2:** Treatment-emergent adverse events.

Adverse events	All grades	Grade 1	Grade 2	Grade 3	Grade 4	Grade 5
CRS grade	27	14	5	3	4	1
CRS, specific symptoms						
Fever	27	14	5	3	4	1
Hypotension	11	0	4	2	4	1
Hypoxemia	3	0	1	1	1	0
Neurotoxicity	1	0	0	0	1	0
Muscle weakness	0	0	0	0	0	0
Nausea	3	1	0	0	2	0
Vomiting	2	0	0	0	2	0
Myalgias	1	1	0	0	0	0
Lung infection	4	1	3	0	0	0
Cerebral hemorrhage	1	0	0	0	1	0
Laboratory abnormalities						
ALT increased	15	6	5	1	2	1
Cr increased	1	0	1	0	0	0
APTT prolonged	11	3	4	1	2	1
Fib decreased	8	2	3	1	2	0

ALT, aminotransferase; Cr, creatinine; APTT, activated partial thromboplastin time; Fib, fibrinogen.

The incidence of CRS after the second infusion was 7/7 (100%). No grade 3–5 CRS and neurotoxicity occurred in these patients.

### Engraftment and GVHD

Ten patients received allo-HSCT after the first hCART19s infusion, with a median time of leukocyte engraftment of 15.5 days (range: 11–25) and platelet engraftment of 20 days (range: 12–36), respectively. All patients obtained a second CR. The incidence of second CR is higher in the CART+HSCT group compared to the CART2 group (100% *vs.* 42.9%, p*=*0.015) (**Figure 1**). One patient experienced grade 1–2 aGVHD, and two patients experienced grade 3–4 aGVHD, with a median time of 40 days (range: 26–45). No patient experienced cGVHD.

### Survival Outcome

**Figure 3:**
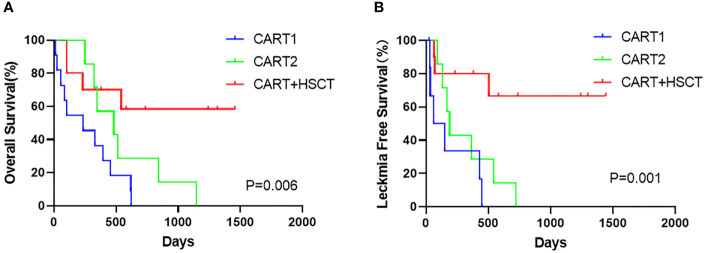
Prognosis of patients after hCART19s therapy. **(A)** The overall survival (OS) of all patients after the infusion of hCART19s according to 3 groups. **(B)** The Leukemia-free survival (LFS) of complete remission (CR) patients according to 3 groups.

At the cutoff (January 1, 2021), the median follow-up time was 1,240 days (95% CI, 709 to 1,770). Among the 10 patients receiving allo-HSCT, the median time from the first hCART19s infusion to transplantation was 63 days. The 1-year OS rates were 70.0% (95%CI, 33.0 to 90.2), 57.1% (95% CI, 17 to 85.1), and 36.4% (95% CI, 11.2 to 62.4) in the CART+HSCT, CART2, and CART1 groups, respectively. Patients in the CART+HSCT group had a higher OS than those in the CART1 group (p*=*0.0063, **Supplementary Figure 1A**) but did not differ from that in the CART2 group (p*=*0.0893, **Supplementary Figure 1B**). The 1-year LFS rates were 80.0% (95%CI 40.9 to 94.6), 28.6% (95% CI, 4.1 to 61.1), and 33.3% (95% CI, 4.6 to 67.6) in the CART+HSCT, CART2, and CART1 groups, respectively. Patients in the CART+HSCT group had a higher LFS than those in the CART1 group (p*=*0.0018) and the CART2 group (p*=*0.0183) (**Supplementary Figures 1C, D**). OS and LFS in the CART1 and CART-2 groups did not differ significantly (p*=* 0.2327, p*=*0.1818, respectively, **Supplementary Figures 1E, F**). When it comes to long-term survival, higher rates of long OS and LFS were achieved in the CART+HSCT group at 3 years compared to the CART2 group (58.3% *vs.* 0, p*<*0.001; 66.7% *vs.* 0, p*<*0.001) (**Figure 3**). Similar results were obtained when the CART+HSCT group was compared to the CART1 group (58.3% *vs.* 0, p*<*0.001; 66.7% *vs.* 0, p*<*0.001) (**Figure 3**).

In addition, three patients relapsed at 30, 44, and 399 days after allo-HSCT. For the CART2 group, the median time to first relapse was 189 days (range: 92 to 719). On the other hand, three patients gained CR after a second hCART19s infusion, of whose median time to second relapse was 92 days (range: 38–120). For the CART1 group, the median time to relapse was 149 days (range: 30–449). Between the first hCART19s infusion and the second infusion, the median relapse time was much shorter after the second infusion (92 *vs.* 178, p*=*0.039) (**Figure 1**). Moreover, one patient relapsed with CD19 negative in the CART1 group and the CART2 group, respectively. All patients who relapsed were CD19 positive after CART+HSCT.

**Figure 4:**
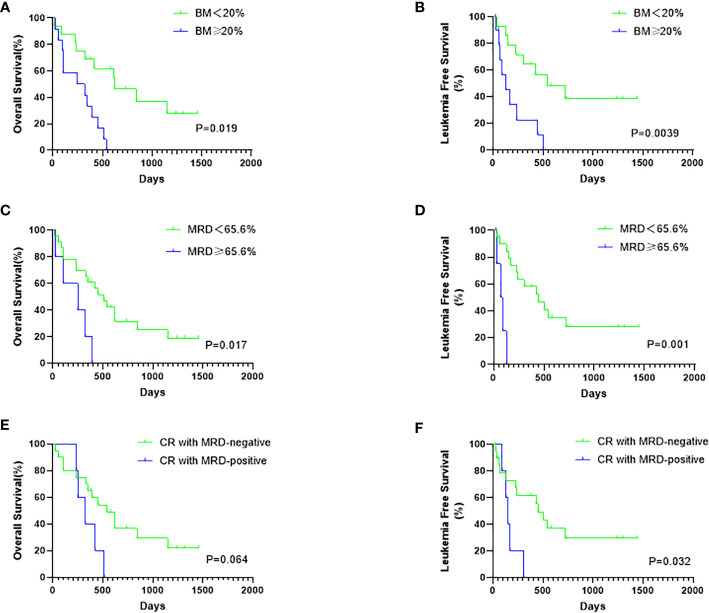
The influence of disease burden at infusion and MRD status after infusion on survival. **(A)** The OS rates of all patients according to the bone marrow (BM) blasts status at infusion. **(B)** The LFS rates of CR patients according to the BM blasts at infusion. **(C)** The OS rates of all patients according to the mRD status at infusion. **(D)** The LFS rates of CR patients according to the MRD status at infusion. **(E)** The OS rates of all 28 patients according to the MRD status after infusion **(F)** The LFS rates of 25 CR patients according to the MRD status after infusion.

Considering all patients possessing positive MRD before hCART19s infusion, we used X-tile to determine the optimal cutoff value for MRD. The MRD cutoff value was with a maximum χ^2^ log-rank value of 5.59 (p*=*0.017) (**Supplementary Figure 2**). Univariate analyses revealed that age, complex chromosome set, recurrence times, treatment times, volume, and the number of infused cells had no significant effects on OS and LFS. The disease burden significantly affected OS and LFS (**Figures 4A–D**). There was also a trend toward better OS (p=0.064) for patients with MRD-negative CR *versus* MRD-positive CR, even when no significant difference was obtained (**Figure 4E**). Patients with MRD-negative CR achieved a longer LFS than those with MRD-positive CR (p=0.032) (**Figure 4F**). Candidate variables with a p value <0.1 on univariate analysis were included in multivariate analysis. In multivariate analyses, the proportion of BM blasts (HR 6.055; 95% CI 1.624–18.933; p*=*0.004) and receiving allo-HSCT (HR 0.250; 95% CI 0.068–0.915; p*=*0.036) were independent predictors for OS. MRD<65.6% at infusion (HR 7.905; 95% CI 1.016-61.505-0.977; p*=*0.048) and receiving HSCT (HR 0.139; 95% CI 0.031–0.619; p*=*0.010) were independent risk factors for LFS. This result showed that receiving HSCT is predictive of both OS and LFS than a second infusion of hCART19s (**Table 3**).

**Table 3:** Multivariate Cox regression analysis for OS and LFS of CR patients (n = 25).

Subgroup	HR	95% CI	*P*
**OS**			
MRD^#^ ≥65.6% *vs* < 65.6%	1.644	0.443-6.099	0.457
BM blasts^#^ ≥20% *vs* <20%	6.055	1.624-18.933	**0.004**
MRD* positive *vs* negative	2.364	0.482-11.602	0.289
Group			0.109
CART1	reference		
CART2	0.556	0.193-1.600	0.276
CART+HSCT	0.250	0.068-0.915	**0.036**
**LFS**			
MRD^#^ ≥65.6% *vs* < 65.6%	7.905	1.016-61.505	**0.048**
BM blasts^#^ ≥20% *vs* <20%	1.950	0.554-6.867	0.310
MRD* positive *vs* negative	1.505	0.273-8.306	0.639
Group			0.031
CART1	reference		
CART2	0.290	0.068-1.235	0.904
CART+HSCT	0.139	0.031-0.619	**0.010**

OS, overall survival; LFS, leukemia-free survival; CART, Chimeric Antigen Receptor T-Cell (CAR-T) therapy; ^#^before CAR-T therapy; *after CAR-T therapy; MRD, minimal residual disease; BM, bone marrow; CART+HSCT group, patients who received allogeneic hematopoietic stem cell transplantation after CAR-T; CART2 group, patients who received a second hCART19s infusion after CAR-T; CART1 group, patients who did not receive HSCT or a second hCART19s infusion.

Bold values, statistical significance.

## Discussion

Recently, results from clinical trials of CD19 CAR-T cell therapy have shown that patients with R/R B-ALL can achieve a high response rate; however, there is a high incidence of early relapse. We retrospectively analyzed the outcomes of patients treated with allo-HSCT or a second CAR-T cell infusion after CAR-T cell therapy, aiming at characterizing the safety and efficacy, and with a goal of identifying an optimal strategy after infusion of CAR-T cells.

The overall response rates of targeted CD19 CAR-T cells are 68%–93% in patients with R/R B-ALL (16, 17). In our study, the CR rate of R/R B-ALL after the first hCART19s infusion was 92.6% (25/27), which was comparable to the results in the previous study. CRS and CRES remain the major adverse events. Consist with early clinical trials, of whose incidence of severe CRS ranged from 26.67% (8/30) to 43.75% (7/16), the incidence of severe CRS after the first hCART19s infusion in our study were seven cases (25%). Only one (2.6%) developed CRES, and the clinical manifestation was epilepsy. The patient’s symptoms were controlled after treatment with dexamethasone and antiepileptics. Some studies have shown that glucocorticoids may affect the efficacy of CAR-T cells and inhibit the proliferation of CAR-T cells. However, other studies reported the opposite results. Therefore, it is necessary to further explore whether glucocorticoids affect the efficacy of CAR-T cells.

Relapse remains a major obstacle after CAR-T cell therapy (18). Anagnostou et al. (19) and Lee et al. (17) reported recurrence rates of 43%–55% in patients achieving CR within 1 year after CAR-T cell treatment. The results of this study showed that the relapse rate in patients who did not receive allo-HSCT was 93.3% (14/15). Relapse can be divided into CD19 positive relapse and CD19 negative relapse, which may be attributed to CAR-T cell exhaust, loss, or mutation of target antigen. The possible mechanisms include growth of CD19-negative cells, lineage switching, cellular gnawing, increased expression of (progressed death) PD-1 in leukemia cells, etc. Seven patients who relapsed after the first hCART19s infusion received a second hCART19s infusion. We identify statistically significant differences in CR rates between the first infusion and the second infusion, suggesting that the second infusion with CAR-T cells had less efficacy. Moreover, the median relapse time was much shorter after the second hCART19s infusion (92 *vs.* 178, p*=*0.039). This result suggests that the second infusion had less efficacy to obtain remission again and failed to achieve durable CR.

It remains to be clarified whether patients may benefit from allo-HSCT after CAR-T cell therapy (8, 16, 20, 21). Consistent with the findings of Hay et al. (8) and Jiang et al. (21), our results supported the fact that a consolidative allo-HSCT after CD19 CAR-T-cell therapy may prolong OS and LFS in patients with R/R B-ALL. Specifically, 10 patients sequentially received allo-HSCT during the CR stage after the first hCART19s infusion, 6 of whom obtained long-term survival, with 1 patient’s survival time reaching 4 years. It is well known that CAR-T cell therapy could achieve deep remission (MRD negative), potentially reverse chemotherapy resistance, and overcome adverse molecular genetic prognosis. However, there was still some recurrence. Moreover, HSCT has the effect of graft-*versus*-leukemia (GVL), which could act as a cure for B-ALL, especially for patients with MRD-negative status before HSCT. In our study, the 1-year OS rates were 70.0%, 57.1%, and 36.4% in the CART+HSCT, CART2, and CART1 groups, respectively. For long-term follow-up, in our CAR-T+HSCT group, the OS rate at 3 years was 58.3% for patients with R/R B-ALL. Since we did not conduct HSCT only when patients were R/R B-ALL, we compared the data from other centers. Duval et al. (22) reported that the OS rate at 3 years was 16% for patients with R/R ALL. Moreover, the mortality rate at 100 days after transplantation was 41% in ALL. Okamoto et al. (23) reported a 3-year OS of 22% in children and adolescents with nonremission ALL. We believe that combined CAR-T cell therapy and HSCT has a synergistic effect. Our data strongly suggest that allo-HSCT is needed to improve the durability of responses after CAR-T therapy. This was a preliminary retrospective clinical study, in which the treatment after the first CAR-T infusion was mainly determined by the disease state and willingness of patients, and thus there was a certain bias. Now, a prospective study of random control trial is warranted based on the clinical study.

In addition, the median time of neutrophil and platelet engraftment was 15.5 days (range: 11–25) and 20 days (range: 12–36), respectively; there was no significant prolongation compared with Luznik’s study (24). At the same time, there was no increase in the incidence of aGVHD (30%) or extensive cGVHD (0%). These results suggest that bridging allo-HSCT after CAR-T cells infusion does not increase the risk of transplantation-related complications or the mortality related to transplantation.

There are also some shortcomings in this study. For example, the number of cases is small, and it is not a prospective study. The timing of transplantation can be further optimized. Further studies with more patients are needed to be conducted in the future. We recognized that it is possible that there is a survival advantage for HSCT because of a selection bias, which could skew the survival analysis in favor of the patients who got HSCT since the HSCT group would be selected for patients who had at least a frank relapse-free survival that exceeded the time to HSCT. Overall, the results of this study demonstrate that CAR-T therapy bridging to HSCT is a feasible, safe, and effective treatment for patients with R/R B-ALL.

## Data Availability Statement

The raw data supporting the conclusions of this article will be made available by the authors, without undue reservation.

## Ethics Statement

The study protocol was approved by the human studies review board at the Affiliated Hospital of Xuzhou Medical University (ClinicalTrials.gov # NCT02782351). The clinical investigation was conducted according to the principles of the Declaration of Helsinki. All patients provided signed informed consent before the hCART19s therapy and allo-HSCT.

## Author Contributions

WS, JZ and KX designed the research; WC, YM, ZS, HC, and RM collected the data and designed the figures. DY, MS, XW, XS, CS, JC, HC, FZ, HS, DL and ZL analyzed and interrupted the results; WC wrote the first draft of manuscript. WS, WC, YM and ZS wrote the revised manuscript. All authors contributed to the article and approved the submitted version.

## Funding

This work was supported by Natural Science Foundation of Jiangsu Province (BK20171181 to WS and BK20161177 to WC); Key Young Medical Talents of Jiangsu Province (QNRC2016791 to WS); Social Development Key Project of Jiangsu Science and Technology Department (BE2019638 to WS.); Project of the Jiangsu Provincial Health and Family Planning Commission/international (JSH-2017-008 to WC); China Postdoctoral Science Foundation project (2016M590507 and 2018T110557 to WC); Xuzhou clinical Backbone Training Project (2018GG006 to WC).

## Conflict of Interest

The authors declare that the research was conducted in the absence of any commercial or financial relationships that could be construed as a potential conflict of interest.

## Publisher’s Note

All claims expressed in this article are solely those of the authors and do not necessarily represent those of their affiliated organizations, or those of the publisher, the editors and the reviewers. Any product that may be evaluated in this article, or claim that may be made by its manufacturer, is not guaranteed or endorsed by the publisher.
