# Supplementary material for: Humanized Anti-CD19 CAR-T Cell Therapy and Sequential Allogeneic Hematopoietic Stem Cell Transplantation Achieved Long-Term Survival in Refractory and Relapsed B Lymphocytic Leukemia: A Retrospective Study of CAR-T Cell Therapy
Source: Front Immunol. 2021 Oct 29;12:755549. doi: 10.3389/fimmu.2021.755549 (PMC8586453; doi:10.3389/fimmu.2021.755549)
Supplement: Supplementary file 1 [file DataSheet_1.docx]

**Supplementary Table 1.** The characteristics of patients

| **No.** | **Diagnosis** | **Gender** | **Age (yr)** | **Complex karyotype (Y/N)** | **MRD (%)** | **BM blasts** | **Number of previous therapies** | **Number of relapses** | **Transduction efficiency of T cells (**%) | **Total CAR-T cells infused (×10^6^/kg)** | **Severe CRS (Y/N)** |
| --- | --- | --- | --- | --- | --- | --- | --- | --- | --- | --- | --- |
| 1 | B-ALL (relapsed) | Male | 39 | N | 7 | 18 | 12 | 3 | 66 | 1 | Y |
| 2 | B-ALL (relapsed) | Female | 57 | N | 49.2844 | 0 | 16 | 1 | 30 | 1 | N |
| 3 | B-ALL (relapsed) | Female | 11 | N | 76.35 | 92 | 13 | *1* | 63 | 1 | N |
| 4 | B-ALL (refractory) | Female | 33 | N | 29.665 | 45 | 2 | - | 35 | 1 | N |
| 5 | B-ALL (relapsed) | Female | 39 | N | 27.9428 | 15 | 6 | 1 | 29 | 1 | N |
| 6 | B-ALL (relapsed) | Male | 14 | Y | 30.784 | 29 | 3 | *1* | 23 | 1 | N |
| 7 | B-ALL (refractory) | Female | 23 | N | 0.1108 | 0 | 3 | - | 20 | 1 | Y |
| 8 | B-ALL (relapsed) | Female | 5 | N | 17.7053 | 36 | 6 | 3 | 21 | 1 | Y |
| 9 | B-ALL (relapsed) | Female | 70 | N | 2.3571 | 11 | 2 | 1 | 20 | 1 | N |
| 10 | B-ALL (relapsed) | Female | 40 | N | 96.9932 | 75 | 5 | 3 | 30 | 1 | Y |
| 11 | B-ALL (relapsed) | Male | 21 | N | 31.5 | 4 | 14 | 1 | 57 | 1 | N |
| 12 | B-ALL (relapsed) | Male | 8 | N | 72.21 | 82 | 7 | 2 | 79.8 | 1 | N |
| 13 | B-ALL (relapsed) | Male | 6 | N | 68.7621 | 42 | 9 | 1 | 59.8 | 1 | N |
| 14 | B-ALL (relapsed) | Female | 7 | N | 24.16 | 86 | 17 | 1 | 65 | 1 | N |
| 15 | B-ALL (refractory) | Female | 68 | N | 36.7 | 95 | 2 | - | 16 | 1 | N |
| 16 | B-ALL (relapsed) | Female | 68 | N | 10 | 2 | 2 | 1 | 25 | 1 | N |
| 17 | B-ALL (relapsed) | Male | 53 | N | 37.08 | 0 | 3 | 1 | 18.8 | 1 | N |
| 18 | B-ALL (relapsed) | Male | 7 | N | 10.42 | 0 | 11 | 1 | 85.8 | 1 | N |
| 19 | B-ALL (relapsed) | Male | 20 | Y | 71.85 | 80 | 3 | 1 | 80.7 | 1 | Y |
| 20 | B-ALL (refactory) | Male | 6 | N | 13.3 | 18 | 2 | - | 65 | 1 | N |
| 21 | B-ALL (relapsed) | Female | 15 | N | 18.1 | 16 | 2 | 1 | 24.5 | 1 | N |
| 22 | B-ALL (relapsed) | Male | 9 | N | 0.03 | 0 | 8 | 2 | 50 | 1 | N |
| 23 | B-ALL (relapsed) | Male | 26 | N | 65.6 | 78 | 11 | 1 | 31 | 1 | N |
| 24 | B-ALL (relapsed) | Female | 41 | N | 24.43 | 86 | 7 | 1 | 70 | 1 | Y |
| 25 | B-ALL (relapsed) | Female | 17 | N | 49.65 | 12 | 5 | 1 | 35 | 1 | Y |
| 26 | B-ALL (relapsed) | Male | 22 | Y | 0.167 | 0 | 3 | 2 | 21 | 1 | N |
| 27 | B-ALL (relapsed) | Female | 54 | N | 0.408 | 0 | 7 | 1 | 45 | 1 | Y |
| 28 | B-ALL (relapsed) | Female | 12 | N | 0.803 | 0 | 2 | 1 | 36 | 1 | N |

Notes: B-ALL =Acute B cell lymphoblastic leukemia; MRD = minimal residual disease; BM= bone marrow; Y= yes; N=no

**Supplementary Table 2.** Patient's treatment flow and clinical responses.

| No. | Group | Date of first T-cell collection | Date of first infusion | Response after first infusion | Relapse after first infusion | Date of second T-cell collection | Date of second infusion | Response after reinfusion | Relapse after reinfusion | Date of BMT | Response afer HSCT | Relapse after HSCT |
| --- | --- | --- | --- | --- | --- | --- | --- | --- | --- | --- | --- | --- |
| 1 | CART1 | 2016/8/1 | 2016/8/22 | NA | ^/^ | / | / | / | / | / | / | / |
| 2 | CART1 | 2016/9/8 | 2016/10/17 | CR, MRD- | CD19+ | / | / | / | / | / | / | / |
| 3 | CART1 | 2016/10/7 | 2016/11/1 | CR, MRD- | CD19+ | / | / | / | / | / | / | / |
| 4 | CART1 | 2017/7/17 | 2017/8/7 | NR | / | / | / | / | / | / | / | / |
| 5 | CART1 | 2017/11/10 | 2017/12/6 | NR | / | / | / | / | / | / | / | / |
| 6 | CART1 | 2017/12/21 | 2018/1/17 | CR, MRD- | CD19+ | / | / | / | / | / | / | / |
| 7 | CART1 | 2018/12/13 | 2019/1/8 | CR, MRD+ | CD19+ | / | / | / | / | / | / | / |
| 8 | CART1 | 2019/2/18 | 2019/3/7 | CR, MRD- | CD19+ | / | / | / | / | / | / | / |
| 9 | CART1 | 2019/8/29 | 2019/9/23 | CR, MRD- | CD19- | / | / | / | / | / | / | / |
| 10 | CART1 | 2020/6/5 | 2020/7/3 | CR, MRD- | CD19+ | / | / | / | / | / | / | / |
| 11 | CART1 | 2016/10/5 | 2016/11/1 | CR, MRD- | CD19+ | / | / | / | / | / | / | / |
| 12 | CART2 | 2016/6/1 | 2016/6/24 | CR, MRD+ | CD19+ | 2016/10/23 | 2016/11/5 | / | / | / | / | / |
| 13 | CART2 | 2016/9/22 | 2016/11/1 | CR, MRD+ | CD19+ | 2017/2/2 | 2017/2/17 | CR | CD19- | / | / | / |
| 14 | CART2 | 2016/11/25 | 2016/12/19 | CR, MRD+ | CD19+ | 2017/6/25 | 2017/7/5 | CR | CD19+ | / | / | / |
| 15 | CART2 | 2018/7/3 | 2018/7/20 | CR, MRD- | CD19+ | 2019/2/13 | 2019/3/5 | / | / | / | / | / |
| 16 | CART2 | 2017/1/8 | 2017/2/10 | CR, MRD- | CD19+ | 2018/8/9 | 2018/8/23 | / | / | / | / | / |
| 17 | CART2 | 2016/3/23 | 2016/4/13 | CR, MRD- | CD19+ | 2018/4/5 | 2018/4/15 | / | / | / | / | / |
| 18 | CART2 | 2016/5/20 | 2016/6/23 | CR, MRD+ | CD19+ | 2016/10/15 | 2016/11/10 | CR | CD19+ | / | / | / |
| 19 | CART+HSCT | 2016/8/18 | 2016/9/14 | CR, MRD- | / | / | / | / | / | 2016/10/30 | CR | CD19+ |
| 20 | CART+  HSCT | 2016/11/28 | 2017/1/4 | CR, MRD- | / | / | / | / | / | 2017/5/21 | CR | CD19+ |
| 21 | CART+  HSCT | 2017/5/16 | 2017/5/27 | CR, MRD- | / | / | / | / | / | 2017/6/28 | CR | CD19+ |
| 22 | CART+  HSCT | 2017/7/24 | 2017/8/10 | CR, MRD- | / | / | / | / | / | 2017/9/26 | CR | CD19+ |
| 23 | CART+  HSCT | 2018/1/12 | 2018/2/12 | CR, MRD- | / | / | / | / | / | 2018/5/28 | CR | CD19+ |
| 24 | CART+  HSCT | 2018/4/4 | 2018/5/7 | CR, MRD- | / | / | / | / | / | 2018/7/9 | CR | CD19+ |
| 25 | CART+  HSCT | 2018/12/13 | 2018/12/26 | CR, MRD- | / | / | / | / | / | 2019/2/27 | CR | CD19+ |
| 26 | CART+  HSCT | 2019/5/5 | 2019/5/31 | CR, MRD- | / | / | / | / | / | 2019/7/31 | CR | CD19+ |
| 27 | CART+  HSCT | 2019/11/20 | 2019/12/9 | CR, MRD- | / | / | / | / | / | 2020/4/8 | CR | CD19+ |
| 28 | CART+  HSCT | 2019/11/27 | 2019/12/18 | CR, MRD- | / | / | / | / | / | 2020/4/15 | CR | CD19+ |

Notes: CART=Chimeric Antigen Receptor T-Cell (CAR-T) therapy; CART+HSCT group=patients who received allogeneic hematopoietic stem cell transplantation after CAR-T; CART2 group=patients who received a second hCART19s infusion after CAR-T; CART1 group=patients who did not receive HSCT or a second hCART19s infusion; MRD=minimal residual disease; BMT=bone marrow transplantation.

**Supplementary Figure 1.** Prognosis of patients after hCART19s therapy. (A) The overall survival (OS) in CART1 and CART+HSCT group. (B) The OS in CART2 and CART+HSCT group. (C) The Leukemia- free survival (LFS) in CART1 and CART+HSCT group. (D) The LFS in CART2 and CART+HSCT group. (E) The OS in CART1 and CART2 group. (F) The LFS in CART1 and CART2 group.


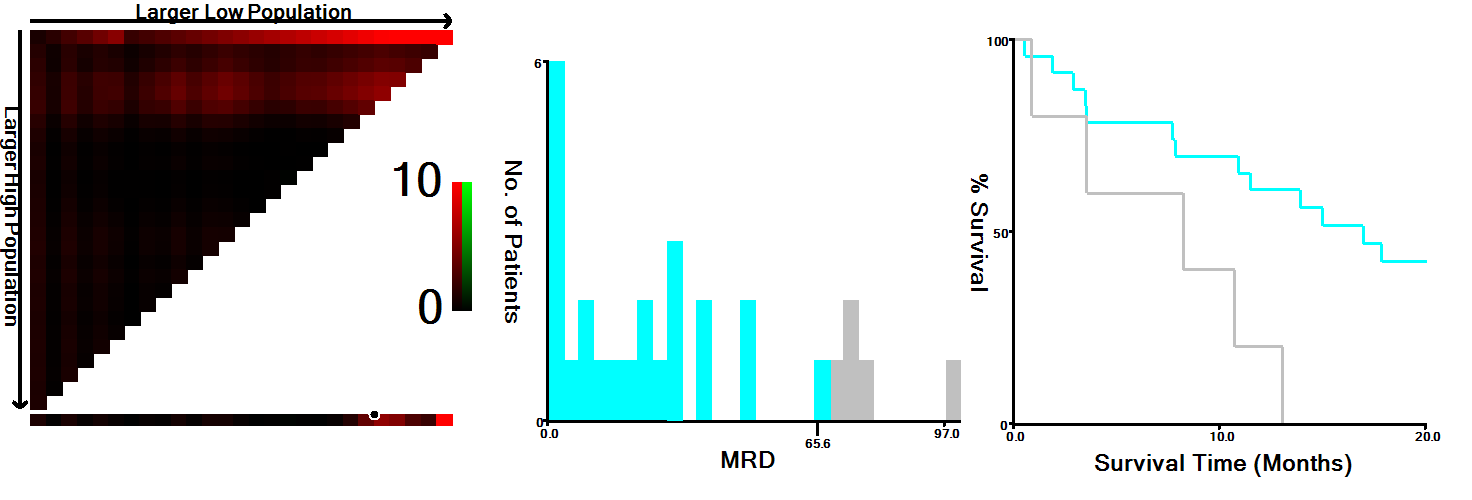


**Supplementary Figure 2.** The optimal cut-point highlighted by the black circle in the left panels is shown on a histogram of the entire cohort (middle panels), and a Kaplan-Meier plot (right panels). P values were determined using the cutoff point defined in all the patients. This figure shows the optimal cutoff point for the MRD (65.6%, χ2 = 5.59, P = 0.017).
